# Impacts of Working Hours, Wages, and Regular Employment Opportunity on Suicide Mortalities of Employed and Unemployed Individuals before and during the COVID-19 Pandemic in Japan

**DOI:** 10.3390/ijerph21040499

**Published:** 2024-04-18

**Authors:** Ryusuke Matsumoto, Eishi Motomura, Motohiro Okada

**Affiliations:** Department of Neuropsychiatry, Division of Neuroscience, Graduate School of Medicine, Mie University, Tsu 514-8507, Japan; matsumoto-r@clin.medic.mie-u.ac.jp (R.M.); motomura@clin.medic.mie-u.ac.jp (E.M.)

**Keywords:** suicide, COVID-19, Japan, social standing, working hours, wages

## Abstract

Standardized suicide mortality rates per 100,000 population (SMRs) in Japan consistently decreased from 2009–2019, but these decreasing trends were reversed to increase in 2020. To clarify the mechanisms of recent increasing suicide in Japan, temporal fluctuations of SMRs disaggregated by sex and employment status (employed and unemployed individuals) and labor indices such as working hours, wages, and regular employment opportunity index (REO) from January 2012 to June 2023 were analyzed using interrupted time-series analysis. Additionally, temporal causalities from labor indices to SMRs were analyzed using vector autoregressive and non-linear auto-regressive distributed lag analyses. Decreasing trends among employed SMRs of both sexes were attenuated after the enactment of the “Work Style Reform Program” in 2018, but male SMRs were unaffected by the COVID-19 pandemic. However, female employed SMRs sharply increased, synchronized with the “Work Style Reform Act” and the COVID-19 pandemic outbreak (the COVID-19 impact was greater than the “Work Style Reform Act”). Additionally, unemployed SMRs of both sexes sharply increased with the revision and scale-down of countermeasures against economic deterioration caused by COVID-19 (“revision of economic supportive countermeasures against economic deterioration caused by COVID-19”). Unexpectedly, after enacting the “Work Style Reform Act”, wages decreased due to possibly decreasing working hours. Increasing REO, which consistently increased, was a protective factor for male suicides, but unemployed SMRs were not affected by any labor indices. It has been established that controlling a heavy workload plays an important role in suppressing the deterioration of physical and mental conditions, including suicide; however, this study suggested that, at least within appropriate ranges of working hours, decreasing working hours due to excessive management probably contributes to increasing suicides of some vulnerable individuals via de-creasing their wages. Although governmental welfare and economic support measures had to be revised according to rapidly changing situations during the COVID-19 pandemic, this study also suggested that temporal gaps among a part of revisions of several welfare and economic support measures were unexpectedly involved in drastically/sharply increasing suicides of unemployed individuals in 2022.

## 1. Introduction

Suicide is the leading cause of age-standardized years of life lost in the global burden of disease in the high-income Asia Pacific region [1]. Globally, suicide had decreased in this century; however, the decreasing trends in suicides had shown signs of reversing after the 2010s [1,2,3]. In the face of global public health problems, suicide has been recognized as a critical public health issue by the World Health Organization in its “Comprehensive Mental Health Action Plan” [4]. This plan contains a target to decrease global suicide mortality by 10% from 2012 to 2020 [4]. Japan has successfully decreased standardized suicide mortality rates per 100,000 population (SMRs) by approximately 20% from 21.99 (2012) to 16.58 (2020) [5,6,7,8,9], whereas these consistently decreasing trends of suicide mortality from 2009 to 2019 in Japan reversed increasing from 2020 [10,11,12,13,14]. The majority of studies concluded that this increasing suicide mortality from 2020 in Japan was possibly caused by the COVID-19 pandemic, since the onset of the reversed increase in suicides in Japan was synchronized with the COVID-19 pandemic outbreak. However, the actual causes of the recent increase in suicide in Japan remain to be clarified. Recently, several studies reported that the decreasing trends of SMRs in Japan had already attenuated since 2018 [10,11,12,13,15,16]. The actual cause underlying the attenuation of decreasing trends in Japan in 2018 has also remained to be clarified.

Suicide is considered to be composed of complexes among multifaceted factors with various interrelated and temporally biopsychosocial determinants at the individual and societal levels [17,18,19]. It has been well known that East Asian countries have higher standardized suicide mortality rates (SMRs), lower male/female suicide ratios, and larger roles in socioeconomic factors compared with Western countries [1,20]. Among individual and social factors for suicide, socioeconomic factors have been established to be prominent suicidal risks. Socioeconomic factors at both the individual and social levels have been intrinsically linked and mutually reinforcing in East Asia. Socioeconomic factors, including unemployment, have been associated with suicide among East Asian males (including those from China, Taiwan, Japan, Korea, and Hong Kong) and have been interpreted in the context of traditional values due to collective rather than individual values, including the importance of maintaining family [20,21,22,23]. In other words, the traditional work-life balance may play an important role in the specific features of suicide in East Asian regions. Although unemployment has been established as one of the major risks for suicide, the recent implementation of generous unemployment supportive measures in high-income countries has contributed to reducing the impact of unemployment as a suicide risk factor [24,25,26,27,28].

In East Asian countries, including Japan, a substantially and rapidly decreasing labor force due to increasing life expectancy and decreasing birthrates became a severe socioeconomic issue [29,30,31]. The increasing retired population contributes to not only decreasing income taxes but also puts pressure on financial expenditures for various welfares, such as pensions and medical expenses [32]. Additionally, the decreasing labor force is a major global public health concern that can be seeding for overwork, which leads to deteriorations of physical and mental health, resulting in increased suicides [33,34]. In order to deal with the decreasing labor force, the Japanese government enacted the “Work Style Reform Act” to revise eight labor-related acts in July 2018 [31]. The “Work Style Reform” program was implemented to improve the following: working environments for both regular and non-regular employees by tightening the upper limit of long working hours and overwork; promotion of increasing participation in paid employment for females and individuals over 65 years; and wage systems independent of the types of employment [31]. Although it is difficult to predict that the “Work Style Reform Act” itself directly attenuated decreasing trends of suicides, since the “Work Style Reform Act” was implemented to improve employment envelopments, the temporal coincidence between the “Work Style Reform Act” enacting and the attenuation of decreasing trends of suicide mortality in Japan from 2018 suggested the existence of some relations or causalities that changing labor indices due to the “Work Style Reform Act” affected suicides.

Although a number of reports during the initial stage of the COVID-19 pandemic were deeply concerned with increasing suicides during the COVID-19 pandemic, major Organization for Economic Co-operation and Development (OECD) countries did not increase suicides during the COVID-19 pandemic [35,36,37,38,39,40,41]. Several reports suggested that the mechanisms of these discrepancies were that timely, generous governmental supportive countermeasures against economic and social deteriorations caused by the COVID-19 pandemic contributed to the prevention of suicides [42,43,44]. Similar to other OECD countries, the Japanese government also provided various economic support countermeasures against economic deteriorations caused by the COVID-19 pandemic, such as the “Sustainability Benefit” [45]. In December 2021, these government supportive countermeasures were revised to economic supports for business revitalization for the post-COVID-19 era, such as from “Sustainability Benefit” to “Business Revitalization Support Fund” [45]. Therefore, analyzing the impacts of this revision of economic supportive countermeasures on suicide mortality is important for the continued infectious pandemic era. According to our hypothesis, to clarify the impacts of these three events, such as the “Work Style Reform Act”, the COVID-19 pandemic outbreak, and the “revision of economic supportive countermeasures against economic deterioration caused by COVID-19”, on suicides, this study determined the temporal fluctuation of age-standardized suicide death rates (SDRs) and SMRs of employed and unemployed individuals disaggregated by the “Work Style Reform Act”, COVID-19, and the “revision of economic supportive countermeasures against economic deterioration caused by COVID-19” were analyzed using interrupted time-series analysis with robust standard error (ITSA). Additionally, temporal causalities from labor indices, including working hours, wages, and regular employment opportunity (REO), published in the “Monthly Labor Survey” in the “Ministry of Health, Labor and Welfare” (MHLW), to suicide mortality were analyzed using vector-autoregressive analysis with Granger causality and robust standard errors (VAR) and non-linear autoregressive distributed lag analysis (NARDL).

## 2. Materials and Methods

### 2.1. Data Source

Monthly suicide numbers disaggregated by sex, age, and employment status (employed and unemployed individuals) were obtained from the “Basic Data on Suicide in the Region” (BDSR) published by the Ministry of Health, Labor and Welfare (MHLW) [46]. Populations disaggregated by sex and age were obtained from the “Surveys of Population, Population Change and the number of households based on the Basic Resident Registration” published by the Ministry of Internal Affairs and Communication (MIAC) [47]. The populations of employed and unemployed individuals were obtained from the “Labor Force Survey” in MHLW [48]. The labor indices, including working hours and wedges disaggregated by employment status (regular and part-time employees), were obtained from the “Monthly Labor Survey” of the MHLW. The labor indices and their abbreviations in the Monthly Labor Survey are summarized in Table 1.

The observation period in this study was January 2012 to June 2023, since the “Monthly Labor Survey” was provided via the Statistics Dashboard of Ministry in MIAC from January 2012 [49], and the COVID-19 pandemic ended in June 2023 in Japan [45,50].

### 2.2. Data Analysis

Monthly standardized suicide mortalities per 100,000 population (SMRs) disaggregated by sex and employment status (employed and unemployed individuals) were calculated by dividing the monthly suicide numbers by the 100,000 population of the corresponding groups in the same period. Monthly age-standardized suicide death rates per 100,000 population (SDRs) of males and females in Japan were adjusted using the Japanese standard age population distribution model in 2015 (2015JSP) [51]. The monthly SDRs and SMRs were then converted to annualized values for 365 days.

The temporal fluctuations, including trends, discontinuities, and their effect sizes on SDRs and SMRs, were analyzed by ITSA using Stata version 17 for Windows (StataCorp, College Station, TX, USA) [12,52,53]. The intervention periods in ITSA were set at July 2018, April 2020, and December 2021, based on the periods of enacting the “Work Style Reform Act”, the COVID-19 pandemic outbreak, and the “revision of economic supportive countermeasures against economic deterioration caused by COVID-19”, respectively [16,31,45]. Symmetric temporal causalities from independent variables (labor indices), including working hours, wages, and the regular employment opportunity index (REO), to SDRs and SMRs were analyzed by VAR using Gretl for Windows v2023c [54]. When the assumption of Granger causality was violated (*p* < 0.05), the sensitivity analysis was conducted using impulse response analysis, which detects the temporal impacts of system shock (increasing one standard deviation of value in a labor index) on target (SDR or employed/unemployed SMR). Lags were analyzed using Akaike information criterion.

Individuals living in an era in which regular employees increase may be numb to the benefits of regular employment or may have adapted to them. However, in an environment in which regular employees decrease, a sudden reversal in REO can have substantial influence on the socioeconomic or psychosocial well-being of individuals. In other words, the relationship between socioeconomic/psychosocial status and the REO may be enhanced when the REO decreases and attenuated when the REO increases. Considering this asymmetry, the asymmetric impacts of the REO on the SDRs/SMRs were analyzed by non-linear autoregressive distributed lag analysis (NARDL) using EViews version 13 for Windows (IHS Markit, London, UK).

This study adhered to the Strengthening the Reporting of Observational Studies in Epidemiology (STROBE) guidelines. The Medical Ethics Review Committee of Mie University waived the requirement for informed consent and ethical approval because the study used data available from publicly accessible government databases.

## 3. Results

The summary of the mean ± standard deviation (SD) between January 2012 and June 2023 is represented in Table 2.

### 3.1. Temporal Fluctuations of SDRs and SMRs

**Figure 1:**
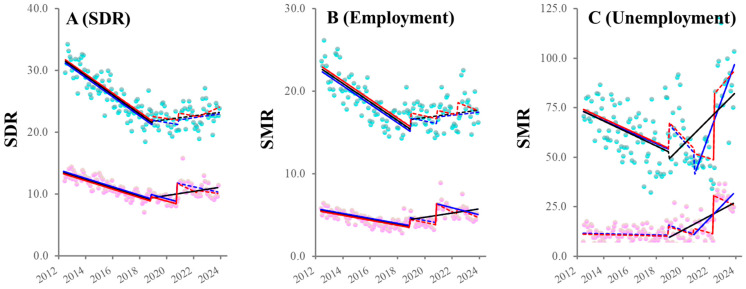
Fluctuation of SDRs and SMRs of employed and unemployed individuals from January 2012 to June 2023 in Japan using ITSA. Panels (**A**–**C**) indicate the trends and discontinuity of SDRs and SMRs of employed and unemployed individuals, respectively. Ordinates indicate the SDR or SMR (per 100,000 population). Abscissas indicate the year. Blue and red circles indicate the observed annualized monthly SDRs and SMRs of males and females, respectively. Black lines indicate the results calculated by ITSA with interventions set during the period of the “Work Style Reform Act” alone. Blue lines indicate the results calculated by ITSA with interventions set during the period of enacting the “Work Style Reform Act” during the COVID-19 pandemic outbreak. Red lines indicate the results calculated by ITSA with interventions set during the period of enacting the “Work Style Reform Act”, the COVID-19 pandemic outbreak, and the “revision of economic supportive countermeasures against economic deterioration caused by COVID-19”. Solid and dotted lines indicate the significant and non-significant trends or discontinuities detected by ITSA, respectively.

SDRs and SMRs of employed and unemployed males indicated decreasing trends before enacting the “Work Style Reform Act”. Decreasing trends in male SDR and employed SMR attenuated after the “Work Style Reform Act” (from significantly decreasing to unchanging) (Figure 1). Neither the COVID-19 pandemic outbreak nor “revision of economic supportive countermeasures against economic deterioration caused by COVID-19” affected trends in SDR and employed SMR (Figure 1). Additionally, the fluctuations of male unemployed SMR indicated complicated fluctuation patterns (Figure 1). When the intervention period was set at the “Work Style Reform Act” alone, male unemployed SMR decreased and increased before and after the “Work Style Reform Act”, respectively. When intervention periods were set at the “Work Style Reform Act” and COVID-19 pandemic outbreaks, male unemployed SMR remained unchanged between the “Work Style Reform Act” and COVID-19 pandemic outbreaks, with a non-statistically positive discontinuity synchronized with the pandemic outbreak, following a drastic increase. When intervention periods were set at the “Work Style Reform Act”, COVID-19 pandemic outbreak, and “revision of economic supportive countermeasures against economic deterioration caused by COVID-19”, male unemployed SMR also remained unchanged between the COVID-19 pandemic outbreak and “revision of economic supportive countermeasures against economic deterioration caused by COVID-19”, with drastically positive discontinuation (a statistically significant sharp increase) synchronized with the “revision of economic supportive countermeasures against economic deterioration caused by COVID-19” (Figure 1).

In females, when the intervention period was set at the “Work Style Reform Act” alone, before the enactment of the “Work Style Reform Act”, trends of female SDR and employed SMR indicated decreasing, but unemployed SMR indicated unchanging (Figure 1). After the “Work Style Reform Act”, all female SDR and SMRs of employed and unemployed individuals increased (Figure 1). Especially, employed SMR sharply increased synchronized with the “Work Style Reform Act” (Figure 1). When intervention periods were set at the “Work Style Reform Act” and COVID-19 pandemic outbreak, between the “Work Style Reform Act” and COVID-19 pandemic outbreak, the decreasing trend of female SDR indicated to remain decreasing, but both SMRs of employed and unemployed individuals did not change (Figure 1). After the pandemic outbreak, the trend of female SDR did not change, but the SMRs of employed and unemployed individuals decreased and increased, respectively (Figure 1). Especially female SDR and employed SMR sharply increased, synchronized with the “Work Style Reform Act” and the pandemic outbreak (Figure 1). When intervention periods were set at the “Work Style Reform Act”, COVID-19 pandemic outbreak, and “revision of economic supportive countermeasures against economic deterioration caused by COVID-19”, all female SDR and SMRs of employed and unemployed individuals indicated unchanging after the COVID-19 pandemic and “revision of economic supportive countermeasures against economic deterioration caused by COVID-19” (Figure 1). Especially female unemployed SMR indicated a drastically positive discontinuity synchronized with the “revision of economic supportive countermeasures against economic deterioration caused by COVID-19” (Figure 1).

### 3.2. Temporal Fluctuations of Working Indices

**Figure 2:**
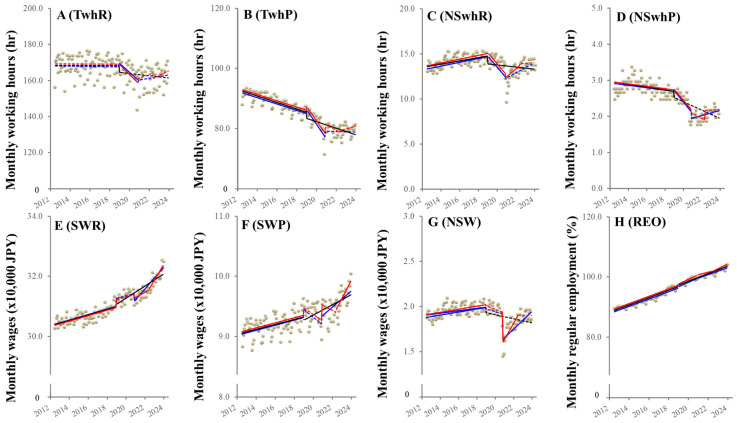
Fluctuation of labor indices in Japan from January 2009 to June 2023 using ITSA. Upper side panels (**A**–**D**) indicate the trends and discontinuity of total working hours of regular employees (TwhR: (**A**)) and part-time employees (TwhP: (**B**)), non-scheduled working hours (overtime working) of regular employees (NSwhR: **C**) and part-time employees (NSwhP: (**D**)), respectively. In the lower side panels, panels (**E**–**G**) indicate the trends and discontinuity of wages: fixed wage for scheduled working hours of regular employees (SWR) and part-time employees (SWP) and wage for non-scheduled working hours (NSW), respectively. Panel (**H**) indicates the trend and discontinuity of regular employment opportunities (REO). Ordinates indicate the monthly working hours (h) in panels (**A**–**D**), monthly wages (JPY × 10,000) in panels (**E**–**G**), and REO (%) in panel (**H**). Abscissas indicate the year. Circles indicate the observed values of monthly labor indices. Black lines indicate the results calculated by ITSA with interventions set during the period of enacting the “Work Style Reform Act” alone. Blue lines indicate the results calculated by ITSA with interventions set during the period of enacting the “Work Style Reform Act” during the COVID-19 pandemic outbreak. Red lines indicate the results calculated by ITSA with interventions set during the period of enacting the “Work Style Reform Act”, the COVID-19 pandemic outbreak, and “revision of economic supportive countermeasures against economic deterioration caused by COVID-19”. Solid and dotted lines indicate the significant and non-significant trends or discontinuities detected by ITSA, respectively.

Before the “Work Style Reform Act”, both total and non-scheduled working hours of part-time employees (TwhP and NSwhP) decreased, whereas the total (TwhR) and non-scheduled working hours (NSwhR) of regular employees did not change and increased, respectively (Figure 2). All working hour indices decreased after the “Work Style Reform Act” (Figure 2). After the pandemic outbreak, the non-scheduled working hours of regular employees (NSwhR) increased, while others shifted to unchanging. After the “revision of economic supportive countermeasures against economic deterioration caused by COVID-19”, the non-scheduled working hours of part-time employees (NSwhP) sharply increased, but others did not sharply increase (Figure 2).

Unexpectedly, all wages transformed from increasing to unchanging after the “Work Style Reform Act” (Figure 2). Scheduled wages of regular employees (SWR) increased after the pandemic outbreak and the “revision of economic supportive countermeasures against economic deterioration caused by COVID-19”. The scheduled wages of part-time employees (SWP) sharply increased with the pandemic outbreak, but its subsequent trends remained unchanging, whereas after the “revision of economic supportive countermeasures against economic deterioration caused by COVID-19”, they increased. Non-scheduled wages (NSW) sharply decreased with the pandemic outbreak but later recovered, whereas after the “revision of economic supportive countermeasures against economic deterioration caused by COVID-19”, they did not change (Figure 2). The REO consistently increased without being affected by the “Work Style Reform Act”, pandemic outbreaks, or “revision of economic supportive countermeasures against economic deterioration caused by COVID-19” (Figure 2).

### 3.3. Synmetric Temporal Causality from Labor Indices to SDRs/SMRs

Single-factor VAR detected several causalities, from labor indices to SDRs and SMRs (Table 3). The unemployed SMRs displayed causal features that were critically different from those of the other groups, since neither the SMRs of unemployed males nor females were related to any labor indices (Table 3). Among males, increasing working hours of part-time employees, such as total working hours (TwhP) and non-scheduled working hours (NSwhP), were positively related to employed SMR, and increasing total working hours of regular employees (TwhR) and non-scheduled working hours of part-time employees (NSwhP), were positively related to male SDR (Table 2). Increasing wages, such as fixed wages for scheduled working hours of regular and part-time employees (SW, SWR, and SWP), were negatively related to male SDR or employed SMR (Table 3). Notably, increasing REO was related to decreasing male SDR and employing SMR (Table 3). Contrary to males, in females, increasing REO was related to decreasing female SDR but was not related to female SMRs of employed or unemployed individuals (Table 3). Female SDR was also negatively related to the increasing scheduled wage of part-time employees (SWP) and non-scheduled wage (NSW) (Table 3). The SMR of employed females was negatively related to the non-scheduled wages of both regular and part-time employees (NSwhR and NSwhP) and the non-scheduled wage (NSW) (Table 3).

**Figure 3:**
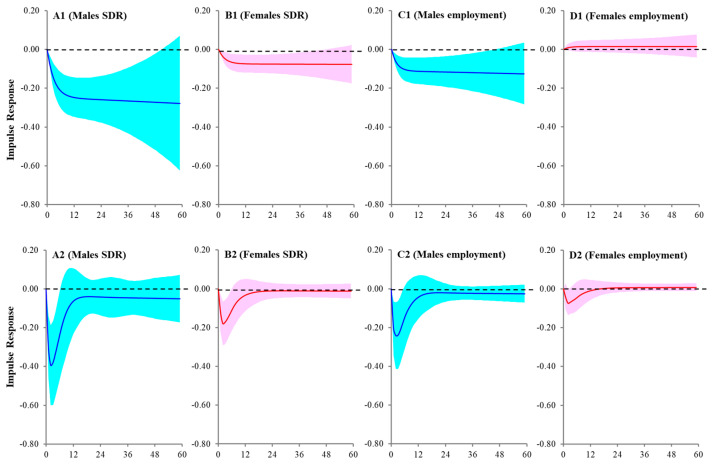
Impulse responses of SDRs and employed SMRs of males and females to increase one SD of REO using VAR. Impulse responses of males SDRs (**A1**,**A2**), males employed SMR (**B1**,**B2**), males employed SMR (**C1**,**C2**), and females employed SMR (**D1**,**D2**) to increase one SD of REO. Upper side panels (**A1**–**D1**) indicated the results using single-factor VAR (REO alone). Lower side panels (**A2**–**D2**) indicate the results using multiple-factor VAR (Twh, TwhR, TwhP, Swh, NSwhR, NSwhP, SW, SWR, SWP, NSW, and REO). Ordinates indicate the impulse response of SDR or SMR. Abscissas indicate the month after the impulse. The blue and red lines and regions indicate the mean ± 95% confidence interval (CI) of responses from males and females, respectively.

Multiple factor VAR detected causalities from the REO to male SDR and SMRs of employed and unemployed individuals, but other labor indices were not related (Table 4 and Figure 3). Female SDR was negatively related to increasing REO, but neither the SMRs of employed nor unemployed individuals were related to the REO (Table 4 and Figure 3).

### 3.4. Asynmetric Temporal Causality from REO to SDRs/SMRs

Female SDR and SMRs of employed and unemployed individuals were not significantly related to the REO (Table 5). Additionally, male SDR and employed SMR were negatively related to both increasing and decreasing REO, but the SMR of unemployed individuals was not related to the REO (Table 5). The asymmetric impacts of the REO on male SDR and employed SMR were predominantly positive influences of decreasing REO rather than negative influences of increasing REO (Table 5). Therefore, suicides increase and decrease when individuals lose and gain regular employment positions, respectively, whereas increasing SMRs due to losing their positions were predominant compared with decreasing SMRs due to gaining regular employment positions.

## 4. Discussion

This study demonstrated that complicated interactions among the “Work Style Reform” program, the COVID-19 pandemic, and “revision of economic supportive countermeasures against economic deterioration caused by COVID-19” affected temporal fluctuations of suicide mortality in Japan using ITSA, VAR, and NARDL. Overall, female SDRs and employed SMRs were greatly affected by the “Work Style Reform Act” and COVID-19 pandemic compared with those of males, and their greater impacts were observed to be sharply increasing (positive discontinuity). Exceptionally, unemployed SMRs of males and females, which did not relate to the enactment of the Work Style Reform Act” or the COVID-19 pandemic outbreak, drastically drastically/sharply increased after the “revision of economic supportive countermeasures against economic deterioration caused by COVID-19”.

Heavy workload (generally over 100 h per month of overtime) is established as a risk for deterioration of physical and/or mental conditions, including suicide [55,56,57,58]. The “Work Style Reform Act” was enacted to limit or control the heavy workload through tightening the upper limit of long working hours and improving wage systems independent of the types of employment [31]. The “Work Style Reform Act” succeeded in decreasing all types of working hours, including scheduled and non-scheduled working hours of both regular and part-time employees. Between enacting the “Work Style Reform Act” and the COVID-19 pandemic outbreak, the decreasing trends of unemployment rates were not affected by the enactment of the “Work Style Reform Act” (which remained decreasing trends) [12,13]. However, increasing trends in wages were attenuated after enacting the “Work Style Reform Act” against the intention of the “Work Style Reform Act”. Ironically, part-time employees received greater negative influences about wages than regular employees. Decreasing wages and increasing minimum wages are related to increasing and decreasing SMRs of minimum wage earners, respectively [59,60,61,62,63]. Single-factor VAR analysis in this study demonstrated that increasing working hours and wages of regular employees protectively affected SDR and employed SMR among males. Conversely, increasing working hours and wages of part-time employees were protective factors for SDR and employed SMR among females, whereas increasing non-scheduled working hours of part-time employees was unexpectedly related to increasing male SDR and employed SMR. Considering the fact that the majority of part-time employees in Japan are female [12,64], attenuated increasing trends in the wages of part-time employees after enacting the “Work Style Reform Act” might negatively affect some vulnerable female groups. In other words, the possibility cannot be denied that the attenuation of the increasing trend of wages, contrary to the intention of the “Work Style Reform Act”, played any role in attenuating the decreasing trend of female suicide mortality in 2018. The COVID-19 pandemic outbreak increased the scheduled wage for regular employees. Conversely, scheduled wages of part-time employees sharply increased synchronized with the pandemic outbreak, but between the pandemic outbreak and “revision of economic supportive countermeasures against economic deterioration caused by COVID-19”, it indicated non-statistically decreasing trends. Furthermore, non-scheduled wages sharply decreased, synchronized with the pandemic outbreak. Therefore, sharply decreasing non-scheduled wages and not increasing scheduled wages of part-time employees may be related to a sharp increase in female SDR and employed SMRs synchronized with the COVID-19 outbreak and followingly persistent (not recovered). After “revision of economic supportive countermeasures against economic deterioration caused by COVID-19”, scheduled wages of both regular and part-time employees increased without affecting working hours or non-scheduled wages. Although controlling overworking is one of the most important public health issues for improving working environments, the unnecessarily tightening restriction on overtime working within appropriate working hours may lead to economic deterioration for some vulnerable employee groups. Notably, in April 2024, the 2nd “Work Style Reform Act” will be implemented to tighten controls on overtime working hours for the construction and logistic industries, which were not covered by the 1st “Work Style Reform Act” in 2018 [65]. We should continue monitoring to ensure that excessive tightening of regulations will not lead to decreasing wages.

Importantly, REO consistently increased without being affected by the “Work Style Reform Act”, COVID-19, or “revision of economic supportive countermeasures against economic deterioration caused by COVID-19”. Multiple-factor VAR detected that REO was the predominant protective factor among labor indices in male SDRs/SMRs. However, its protective impacts on female SDR could be detected, but on both female employed and unemployed SMRs, they could not be observed. Precarious employees have been increasing worldwide, including in Japan [48,66]. The welfare and wages of precarious employees in Japan were inferior to those of regular employees [67]. Furthermore, precarious employees could not receive support from their supervisors or co-workers, compared with their regular employed peers [68]. Increasing numbers of precarious employees, whose working conditions, wages, and welfare are inferior to those of regular employees, have been considered socioeconomic and psychosocial issues in Japan [69]. Considering the inferior status of non-regular employees, the contribution of increasing regular employment opportunities (REO) to decreasing male SDRs and SMRs is easily understood. However, in this study, NARDL detected an asymmetric influence between increasing and decreasing REO on male SDRs/SMRs. When equivalently increasing after decreasing REO, the decreasing male SDRs/SMRs induced by increasing REO are smaller than the previously increasing SMRs induced by decreasing REO. Conversely, the influence between increasing and decreasing REO on female SDRs/SMRs was almost equal. Therefore, obtaining regular employment opportunities is important [70], and the loss of these opportunities has critical implications for males in Japan. However, without understanding traditional Japanese family circumstances, it is impossible to accurately interpret the underlying basis of the discrepancy in response to regular employment opportunities between males and females.

In Japan, governmental social welfare has provided generous support systems for households rather than individuals through tax exemptions, universal public pensions, and health insurance systems [71]. To benefit from these generous support systems, the majority of households in Japan adjusted the annual incomes of spouses partnered with regular employees to less than JPY 1,300,000 [72]. Therefore, not only traditional Japanese family values but also generous governmental support systems for households play fundamental roles in Japanese-specific household situations, in which males continue to be the main breadwinners of households [71,72,73]. In other words, the head of household continuously acquiring regular employment can help maintain benefits from the generous governmental household support system, resulting in spouses being exculpated from employment pressure; conversely, when heads of household lose their regular employment, this not only reduces household incomes, but households also cannot receive generous governmental household support, thus increasing work pressure on their spouses. These Japanese household situations [71,72,73] provide a plausible interpretation of the reason why the contribution of increasing regular employment opportunity (REO) to decreasing male SDRs/SMRs. In the observation periods, the consistently increasing REO is, at least partially, considered to contribute to the protection of male SDRs/SMRs.

Furthermore, it is well known that male suicide is more sensitive to socioeconomic deterioration than female suicide, whereas female suicides were more sensitive to economic recession in Hong Kong during the Asia Economic Crisis and Japan during the COVID-19 pandemic outbreak, reported as the “gender paradox” [20,74,75]. The increasing female labor force participation, including low-skilled and low-wage women (non-regular workers), has been speculated to be the underlying mechanism of the “gender paradox” [75]. The low-skilled/low-wage worker is usually the most expendable in times of economic recession; as a result of this, women may be as affected as men, if not more so, by deteriorating employment conditions [20,74,75]. In Japan, the labor force participation rates of females during the Asian Financial Crisis (1997–1998) were approximately 60%, but that began increasing in the late 2010s and was more than 75% during the pandemic (those of males were approximately 85% over the same time) [12,13,64,75,76]. It has already been reported that female labor force participation rates contribute to the positive association between CURs and SMRPs over time [77]. Therefore, increasing female labor force participation rates from 1990 to 2020 can explain the discrepancy between insensitivity during the Asian economic crisis and high sensitivity during the early stage of the pandemic to increasing CURs of female SMRPs. Furthermore, the part-time employment rate in Japan is the highest (39.1%) among OECD countries (OECD average: 25.3%), and part-time employees are predominantly female in Japan [12,13,64,75,76].

In December 2021, various government supportive countermeasures against economic deterioration caused by COVID-19, such as the “Sustainability Benefit”, were revised or scaled down to the “Business Revitalization Support Fund” by the “revision of economic supportive countermeasures against economic deterioration caused by COVID-19” [45]. These measures provided direct financial support to mainly small enterprises but not to individuals; however, governmental implementation of support for unemployed individuals related to COVID-19 was continued until May 2023 in the form of “COVID-19 leave support payments and subsidies” [45]. Initially, we expected the impacts of “revision of economic supportive countermeasures against economic deterioration caused by COVID-19” on employed SMRs to be dominant compared with those of unemployed SMRs due to the above background, whereas the detected results made it difficult to interpret that the unemployed SMRs of both males and females were sharply increased by enacting “revision of economic supportive countermeasures against economic deterioration caused by COVID-19”. Recently, MHLW released interesting statistics that suggested the plight of unemployed individuals during COVID-19 was dramatically changing compared with before the COVID-19 pandemic [78]. Before the COVID-19 pandemic, beneficiaries of “Housing Security Benefits”, which is a governmental support program to provide housing (rent for housing) for individuals facing economic hardship, had decreased from 6,613 (in 2015) to 3,972 cases (in 2019) due to enhancing welfare for precarious/non-regular employees (employers provide housing for precarious/non-regular employees) [78], whereas beneficiaries drastically increased in 2020 (134,946 cases) [50,78]. Before the COVID-19 pandemic, the period for receiving housing security benefits was three months. Economic deterioration had not improved due to the prolonged COVID-19 pandemic; as a result, the coverage period of “Housing Security Benefits” was extended to 12 months from January 2021 [78]. Therefore, when individuals facing economic hardship cannot improve their poverty or gain their jobs within 2021, they are at risk of losing not only their unemployment benefits but also their housing in 2022. Although the governmental welfare and economic support measures had to be revised according to changing situations caused by the COVID-19 pandemic, this study suggests that temporal gaps among these political revisions were unexpectedly involved in increasing unemployed SMRs. Additionally, considering the unfavorable socioeconomic/psychosocial situations of unemployed individuals, a revision to “revision of economic supportive countermeasures against economic deterioration caused by COVID-19” might also be perceived as anomic shock [79]. Anyway, increasing unemployed SMRs during the late phase of COVID-19 were actually induced by different factors than general or employed individuals.

Several limitations in this study warrant mention. First, this study analyzed causality from labor indices disaggregated by regular and part-time employees to SDRs/SMRs since the “Monthly Labor Survey” provides labor indices of regular and part-time employees. In Japan, precarious workers include contract, part-time, and dispatched workers, whereas part-time employees are approximately 50% of all precarious employees [31,48,68,73]. Therefore, although this study can comprehensively analyze the impacts of labor indices on the suicides of regular employees, the analysis of the impacts of labor indices on the suicides of non-regular or precarious employees cannot be claimed to be comprehensive. Second, this cross-sectional study is observational and cannot be ignored as being probably subject to the ecological fallacy. Comparisons in suicide mortality (sex and employment status) across different populations over the same time period or within the same population over time are subject to confounding, as information on potential confounder(s) may not be available and associations at the population level do not necessarily represent associations at the individual level (ecological fallacy).

## 5. Conclusions

This study determined the temporal fluctuation of SDRs and SMRs of employed and unemployed individuals and temporal causalities from labor indices (working hours, wages, and REO) to SDRs and SMRs from January 2012 to June 2023 to explore the mechanisms underlying the recent increase in suicide in Japan. The results of this study suggest that the mechanisms underlying the recent increase in suicides in Japan may be composed of at least three elements. Before the COVID-19 pandemic, the suppression of increasing wages for part-time employees via decreasing working hours under the “Work Style Reform Act” did not directly contribute to increasing suicide but possibly did attenuate the decreasing trends of suicide. The COVID-19 pandemic outbreak played an important role in sharply increasing suicides among employed females via sharply decreasing non-scheduled wages. Therefore, the SMR of employed females was relatively sensitive to wages compared with that of males, whereas the SMR of employed males was probably more sensitive to regular employment opportunities than their wages. These discrepancies between SMRs of employed males and females are possibly involved in the employment status (non-regular employees are overwhelmingly females). Conversely, in the late COVID-19 pandemic, the suicides of unemployed males and females, which were insensitive to the “Work Style Reform Act” or pandemic outbreak, sharply increased, synchronized with the “revision of economic supportive countermeasures against economic deterioration caused by COVID-19”, but the suicides of employed males and females did not significantly change. These results suggest that the risk factors of unemployed individuals were quite different from those of employed individuals. Additionally, the changing plight of unemployed individuals during the COVID-19 pandemic probably played an important role in the drastic increase in SMRs of unemployed individuals in 2022.

## Figures and Tables

**Table 1:** Labor indices in the “Monthly Labor Survey”.

**Monthly Working Hours (h):**	
Twh	Monthly total working hours among regular and part-time employees.
TwhR	Monthly total working hours of a regular employee.
TwhP	Monthly total working hours of a part-time employee.
Swh	Monthly scheduled working hours among regular and part-time employees.
NSwhR	Monthly non-scheduled working hours (overtime) of a regular employee.
NSwhP	Monthly non-scheduled working hours (overtime) of a part-time employee.
**Wages (JPY):**	
SW	Monthly fixed wage for scheduled working hours among regular and part-time employees.
SWR	Monthly fixed wage for scheduled working hours of a regular employee.
SWP	Monthly fixed wage for scheduled working hours of a part-time employee.
NSW	Monthly wage for non-scheduled working hours (overworking) among regular and part-time employees.
**Regular Employment Opportunity** **Index**	
REO	Monthly normalized value of the number of regular employees in 2020 is 100.

**Table 2:** Summary of descriptive statistics.

Variable	Abbreviation	Mean ± SD	
Monthly total working hours among regular and part-time employees (hr).	Twh	141 ± 6.0	
Monthly total working hours of a regular employee (hr).	TwhR	166.1 ± 6.4	
Monthly total working hours of a part-time employee (hr).	TwhP	85.4 ± 5.0	
Monthly scheduled working hours among regular and part-time employees (hr).	Swh	130.9 ± 5.5	
Monthly non-scheduled working hours (overtime) of a regular employee (hr).	NSwhR	13.9 ± 0.9	
Monthly non-scheduled working hours (overtime) of a part-time employee (hr).	NSwhP	2.6 ± 0.4	
Monthly fixed wage for scheduled working hours among regular and part-time employees (JPY × 10,000).	SW	24.4 ± 0.3	
Monthly fixed wage for scheduled working hours of a regular employee (JPY × 10,000).	SWR	31.1 ± 0.5	
Monthly fixed wage for scheduled working hours of a part-time employee (JPY × 10,000).	SWP	9.3 ± 0.2	
Monthly wage for non-scheduled working hours (overworking) among regular and part-time employees (JPY × 10,000).	NSW	19.2 ± 1.0	
Monthly normalized value of the number of regular employees in 2020 is 100.	REO	96.1 ± 4.4	
		(male)	(female)
Monthly age-standardized suicide death rates (per 100,000 population).	SDR	24.8 ± 3.5	10.9 ± 1.6
Monthly standardized suicide mortalities of employed individuals (per 100,000 population).	employed SMR	18.2 ± 2.5	5.0 ± 0.9
Monthly standardized suicide mortalities of unemployed individuals (per 100,000 population).	unemployed SMR	63.9 ± 17.0	14.2 ± 7.4

**Table 3:** Temporal causality from labor indices to SDRs and SMRs using single-factor VAR.

	Male						Female					
	AdjR^2^	F	*p*	β	*p*		AdjR^2^	F	*p*	β	*p*	
**SDR**												
**Twh**	0.738	238.354	0.000	−0.030	0.196		0.569	81.390	0.000	0.001	0.944	
**TwhR**	0.743	240.469	0.000	−0.049	0.012	**	0.570	77.515	0.000	−0.010	0.449	
**TwhP**	0.739	233.926	0.000	0.056	0.185		0.573	95.801	0.000	0.023	0.349	
**Swh**	0.738	238.747	0.000	−0.035	0.162		0.569	81.927	0.000	0.005	0.785	
**NSwhR**	0.737	242.562	0.000	−0.152	0.292		0.585	68.144	0.000	−0.001	0.699	
**NSwhP**	0.746	235.189	0.000	1.023	0.031	*	0.570	91.764	0.000	0.119	0.663	
**SW**	0.744	232.332	0.000	−0.115	0.045	*	0.569	81.957	0.000	−0.006	0.870	
**SWR**	0.748	236.407	0.000	−0.093	0.014	*	0.576	95.593	0.000	−0.028	0.205	
**SWP**	0.759	168.024	0.000	−0.143	0.092		0.579	136.305	0.000	−0.072	0.033	*
**NSW**	0.736	240.937	0.000	−0.020	0.874		0.579	76.259	0.000	−0.309	0.015	*
**REO**	0.758	247.187	0.000	−0.180	0.001	**	0.586	151.737	0.000	−0.055	0.013	*
**Employment**												
**Twh**	0.540	81.560	0.000	0.019	0.426		0.377	44.149	0.000	−0.007	0.551	
**TwhR**	0.538	82.289	0.000	−0.001	0.973		0.376	43.780	0.000	−0.005	0.606	
**TwhP**	0.552	75.984	0.000	0.064	0.022	*	0.382	44.400	0.000	−0.016	0.235	
**Swh**	0.540	81.735	0.000	0.020	0.435		0.376	44.107	0.000	−0.004	0.770	
**NSwhR**	0.538	81.289	0.000	−0.031	0.815		0.422	49.523	0.000	−0.247	0.019	*
**NSwhP**	0.553	73.245	0.000	0.820	0.025	*	0.407	46.448	0.000	−0.473	0.006	**
**SW**	0.413	48.664	0.000	0.001	0.525		0.413	48.664	0.000	0.001	0.525	
**SWR**	0.551	81.686	0.000	−0.059	0.024	*	0.387	44.428	0.000	0.020	0.110	
**SWP**	0.548	84.331	0.000	−0.117	0.083		0.380	44.324	0.000	0.030	0.274	
**NSW**	0.539	81.395	0.000	0.067	0.575		0.429	50.215	0.000	−0.232	0.013	*
**REO**	0.563	86.149	0.000	−0.107	0.007	**	0.454	53.262	0.000	0.000	0.493	
**Unemployment**												
**Twh**	0.392	27.265	0.000	−0.272	0.202		0.040	3.957	0.021	0.109	0.056	
**TwhR**	0.394	28.198	0.000	−0.275	0.168		0.029	2.991	0.054	0.069	0.210	
**TwhP**	0.387	26.764	0.000	−0.205	0.376		0.051	4.229	0.017	−0.004	0.663	
**Swh**	0.394	27.689	0.000	−0.325	0.165		0.038	3.670	0.028	0.111	0.075	
**NSwhR**	0.384	30.224	0.000	0.589	0.544		0.032	3.639	0.029	0.541	0.114	
**NSwhP**	0.384	27.773	0.000	−1.292	0.626		0.043	3.119	0.047	1.811	0.096	
**SW**	0.390	27.119	0.000	0.537	0.279		0.020	2.015	0.137	0.001	0.995	
**SWR**	0.390	26.829	0.000	0.289	0.243		0.030	2.285	0.106	−0.090	0.304	
**SWP**	0.383	31.340	0.000	−0.056	0.923		0.024	2.081	0.129	−0.126	0.526	
**NSW**	0.384	31.831	0.000	0.531	0.505		0.033	3.535	0.032	0.491	0.098	
**REO**	0.384	28.980	0.000	0.089	0.749		0.047	3.182	0.045	−0.171	0.092	

The table summarizes the results calculated using vector autoregressive analysis with Granger causality and robust standard errors (VAR) between suicide mortality (SDR or employed/unemployed SMR) and single factor labor index (Twh, TwhR, TwhP, Swh, NSwhR, NSwhP, SW, SWR, SWP, NSW, or REO). Twh: Monthly total working hours among regular and part-time employees; TwhR: Monthly total working hours of regular employees; TwhP: Monthly total working hours of part-time employees; Swh: Monthly scheduled working hours among regular and part-time employees, NSwhR: Monthly non-scheduled working hours of regular employees; NSwhP: Monthly non-scheduled working hours of part-time employees; SW: Monthly fixed wage for scheduled working hours among regular and part-time employees; SWR: Monthly fixed wage for scheduled working hours of regular employees; SWP: Monthly fixed wage for scheduled working hours of part-time employees. NSW: Monthly wage for non-scheduled working hours (overworking) among regular and part-time employees, REO: The monthly normalized value of the number of regular employees in 2020 is 100. AdjR^2^: adjusted R^2^ value, F: F value calculated by VAR, β: coefficient, *p*: probability (significance level), * *p* < 0.05, ** *p* < 0.01. The time lags of variables in VAR are set to one month.

**Table 4:** Temporal causality from labor indices to SDRs and SMRs using multiple-factor VAR.

Male	AdjR^2^	F	*p*		β	*p*		Female	AdjR^2^	F	*p*		β	*p*	
**SDR**	0.805	58.334	0.000	**Twh**	0.154	0.971			0.678	27.684	0.000	**Twh**	−3.719	0.065	
				**TwhR**	−0.573	0.779						**TwhR**	−0.830	0.503	
				**TwhP**	0.073	0.941						**TwhP**	−0.284	0.621	
				**Swh**	0.254	0.952						**Swh**	4.789	0.082	
				**NSwhR**	0.723	0.813						**NSwhR**	3.530	0.070	
				**NSwhP**	−3.236	0.183						**NSwhP**	0.173	0.904	
				**SW**	0.098	0.926						**SW**	0.052	0.931	
				**SWR**	0.403	0.570						**SWR**	0.163	0.708	
				**SWP**	0.246	0.587						**SWP**	0.165	0.492	
				**NSW**	−0.165	0.756						**NSW**	−0.377	0.325	
				**REO**	−1.104	0.001	**					**REO**	−0.436	0.022	*
**Employment**	0.610	18.223	0.000	**Twh**	1.886	0.660			0.489	10.126	0.000	**Twh**	−2.182	0.156	
				**TwhR**	2.490	0.272						**TwhR**	−0.439	0.635	
				**TwhP**	1.093	0.300						**TwhP**	−0.191	0.657	
				**Swh**	−5.762	0.247						**Swh**	2.779	0.171	
				**NSwhR**	−3.874	0.291						**NSwhR**	1.982	0.186	
				**NSwhP**	−3.743	0.149						**NSwhP**	0.134	0.895	
				**SW**	1.644	0.134						**SW**	0.080	0.858	
				**SWR**	−0.863	0.262						**SWR**	0.065	0.838	
				**SWP**	−0.087	0.842						**SWP**	0.055	0.752	
				**NSW**	0.547	0.301						**NSW**	−0.206	0.485	
				**REO**	−0.760	0.017	*					**REO**	−0.159	0.224	
**Unemployment**	0.498	12.385	0.000	**Twh**	−16.525	0.603			0.057	1.703	0.074	**Twh**	5.480	0.682	
				**TwhR**	22.783	0.187						**TwhR**	4.371	0.493	
				**TwhP**	9.936	0.219						**TwhP**	1.499	0.611	
				**Swh**	−18.776	0.610						**Swh**	−11.985	0.473	
				**NSwhR**	−4.901	0.855						**NSwhR**	−5.827	0.622	
				**NSwhP**	−26.342	0.105						**NSwhP**	−1.947	0.794	
				**SW**	13.608	0.098						**SW**	3.551	0.221	
				**SWR**	−6.841	0.247						**SWR**	−2.437	0.245	
				**SWP**	1.025	0.771						**SWP**	−0.422	0.707	
				**NSW**	0.771	0.869						**NSW**	−0.363	0.828	
				**REO**	−6.986	0.001	**					**REO**	−0.084	0.897	

The table summarizes the results calculated using multiple-factor vector autoregressive analysis with Granger causality and robust standard errors (VAR) between suicide mortality (SDR or employed/unemployed SMR) and all factors of labor indices (Twh, TwhR, TwhP, Swh, NSwhR, NSwhP, SW, SWR, SWP, NSW, and REO). Twh: Monthly total working hours among regular and part-time employees; TwhR: Monthly total working hours of regular employees; TwhP: Monthly total working hours of part-time employees; Swh: Monthly scheduled working hours among regular and part-time employees. NSwhR: Monthly non-scheduled working hours (overtime) of regular employees; NSwhP: Monthly non-scheduled working hours (overtime) of part-time employees; SW: Monthly fixed wage for scheduled working hours among regular and part-time employees; SWR: Monthly fixed wage for scheduled working hours of regular employees; SWP: Monthly fixed wage for scheduled working hours of part-time employees; NSW: Monthly wage for non-scheduled working hours (overworking) among regular and part-time employees; REO: The monthly normalized value of the number of regular employees in 2020 is 100. AdjR^2^: adjusted R^2^ value, F: F value calculated by VAR, β: coefficient, *p*: probability (significance level), * *p* < 0.05, ** *p* < 0.01. The time lags of variables in VAR are set to one month.

**Table 5:** Asymmetric analysis of temporal causality from REO to SDRs and SMRs of employed and unemployed individuals of males and females using NARDL.

Males	Adj R^2^	F	*p*		Long (p)		Short (p)		Joint (p)			β	SE	T	*p*	
**SDR**	0.359	16.152	0.000	**	0.036	*	0.001	**	0.000	**	REO(+)	−2.447	1.028	−2.381	0.019	*
											REO(−)	−4.500	2.134	−2.109	0.037	*
**Employment**	0.411	11.217	0.000	**	0.073		0.136		0.011	*	REO(+)	−1.659	0.767	−2.164	0.032	*
											REO(−)	−3.212	1.602	−2.005	0.047	*
**Unemployment**	0.289	5.917	0.000	**	0.074		0.739		0.113		REO(+)	−11.891	6.945	−1.712	0.089	
											REO(−)	−25.286	14.436	−1.752	0.082	
**Females**	**Adj R^2^**	**F**	***p***		**Long (p)**		**Short (p)**		**Joint (p)**			**β**	**SE**	**T**	***p***	
**SDR**	0.183	4.318	0.000	**	0.158		0.307		0.055		REO(+)	−0.803	0.451	−1.782	0.077	
											REO(−)	−1.504	0.933	−1.612	0.109	
**Employment**	0.184	7.073	0.000	**	0.137		0.913		0.300		REO(+)	−0.311	0.235	−1.325	0.188	
											REO(−)	−0.685	0.485	−1.412	0.160	
**Unemployment**	0.218	8.522	0.000	**	0.074		0.739		0.113		REO(+)	−1.505	1.297	−1.160	0.248	
											REO(−)	−4.151	2.679	−1.550	0.124	

The table summarizes the results calculated using non-linear autoregressive distributed lag analysis (NARDL) between suicide mortality (SDR or SMR of employed or unemployed SMR) and REO (the monthly normalized value of the number of regular employees in 2020 as 100). AdjR^2^: adjusted R^2^ value; F: F value calculated by NARDL; *p*: probability (significance level); Long (p): probability of long-run asymmetry in NARDL, Short (p): probability of short-run asymmetry in NARDL; joint (p): probability of combinations of long- and short-run asymmetry in NARDL. REO(+): response to increasing REO; REO(−): response to decreasing REO; β: coefficient, T: T value of REO(+) or REO(−) in NARDL, * *p* < 0.05, ** *p* < 0.01.

## Data Availability

All raw data are publicly available to any person via Japanese national databases, including the “Basic Data on Suicide in the Region” (BDSR) in a national database of the MHLW, the “Surveys of Population, Population Change”, the “Basic Resident Registration”, the “Labor Force Survey”, and the “Monthly Labor Survey” in national databases of the MHLW.
